# Two Cases of Colonic Perforation Following Upper Gastrointestinal Barium Examinations

**DOI:** 10.7759/cureus.74892

**Published:** 2024-12-01

**Authors:** Hideo Kidogawa, Ryo Nonomura, Toshihito Uehara, Shin Shinyama, Kohji Okamoto

**Affiliations:** 1 Department of Surgery, Kitakyushu City Yahata Hospital, Kitakyushu, JPN; 2 Department of Thoracic Surgery, Tohoku Medical Pharmaceutical University, Sendai, JPN

**Keywords:** barium, colonic perforation, gastric cancer screening, peritonitis, upper gastrointestinal series

## Abstract

This report presents two cases of colonic perforation that occurred following an upper gastrointestinal series (UGIS) with barium as part of a health screening. UGIS is a widely performed examination in Japan and is useful for the early detection of gastric cancer and peptic ulcers, but it carries a rare risk of causing serious gastrointestinal perforation. This study details the mechanisms of perforation, risk factors, difficulties in imaging diagnosis, and treatment options. Cases of perforation require early diagnosis and appropriate surgical intervention, with Hartmann's procedure or colostomy being the common choices. This case series emphasizes the need to recognize the potential risks associated with UGIS, even though it is considered relatively safe. With the advancement of endoscopic examination technology in recent years, endoscopy is increasingly being adopted as an alternative to UGIS in health screenings, and there is a need to reconsider future examination methods.

## Introduction

Upper gastrointestinal barium studies are widely performed in Japan as part of health screenings and are valued for their accessibility and ability to detect early-stage abnormalities, such as gastric cancer and peptic ulcers [1]. However, gastrointestinal perforation, though rare (approximately three cases per 1,010,000 examinations), can lead to severe complications, including peritonitis and sepsis, if not promptly treated [2,3]. Identified risk factors include advanced age, diverticulosis, and chronic constipation, which can increase the likelihood of perforation [4,5].

Here, we report two cases of colonic perforation following upper gastrointestinal barium examinations, aiming to highlight the mechanisms and clinical implications of this rare but serious complication.

## Case presentation

Case 1

A 53-year-old female with a history of ventricular septal defect presented to our hospital. Three days prior, she had undergone an upper gastrointestinal barium examination as part of a comprehensive medical check-up. Since the examination, she had noted daily passage of white stool. Subsequently, she developed abdominal pain, which progressively worsened, prompting her to be transported to our hospital on an emergency basis.

The patient was 155.0 cm in height and weighed 55.0 kg. Her body temperature was 35.1°C. Blood pressure was 92/76 mmHg, with a pulse rate of 55 beats per minute. Tenderness was noted in the left abdominal region, but there was no evidence of rebound tenderness or board-like rigidity.

Initial blood biochemical investigations at the time of admission revealed abnormalities in white blood cell count, which was elevated, while C-reactive protein (CRP) levels were within normal limits. Detailed laboratory findings are summarized in Table 1.

**Table 1:** Serum laboratory results on admission and hospital day three WBC: white blood cells; RBC: red blood cells; BUN: blood urea nitrogen; AST: aspartate transferase; ALT: alanine transaminase; INR: international normalized ratio; PT: prothrombin time; APTT: partial thromboplastin time; CRP: C-reactive protein; NA: not assessed

Parameters	On admission	Hospital day 3	Reference Range
WBC	12.1	8.8	3.3-8.6 x 10^3^/uL
Neutrophils relative percent	85.4	90.9	44.0-70.0%
Lymphocytes	10.9	7.4	31.0-49.0%
Monocytes	3.2	1.2	3.0-8.0%
Eosinophils	0.2	0.4	1.0-5.0%
Basophils	0.3	0.1	0.0-3.0%
RBC	4.97	3.66	3.86-4.92 x 10^6^/uL
Hemoglobin	16.0	11.9	11.6-14.8 g/dL
Hematocrit	47.9	34.1	35.1-44.4%
Platelet count	212	118	158-348 x 10^3^/uL
PT	98.0	NA	75-125%
INR	1.0	NA	
APTT	25.4	NA	25-39 seconds
Total Protein	6.1	4.6	6.6-8.1 g/dL
Albumin	3.6	2.2	4.1-5.1 g/dL
Total Bilirubin	0.4	0.4	0.4-1.5 mg/dL
AST	21	19	13-30 U/L
ALT	20	14	7-23 U/L
Alkaline phosphatase	249	168	106-322 IFCC
BUN	14.4	19.7	8-20 mg/dL
Creatinine	0.76	0.76	0.46-0.79 mg/dL
Sodium	142	141	138-145 mmol/L
Potassium	4	4	3.6-4.8 mmol/L
Chloride	108	111	101-108 mmol/L
Calcium	9.0	8.5	8.8-10.1 mg/dL
CRP	0.02	36.95	0.00-0.14 mg/dl
Lactate	NA	1.0	<=2.0 mmol/L

**Figure 1:**
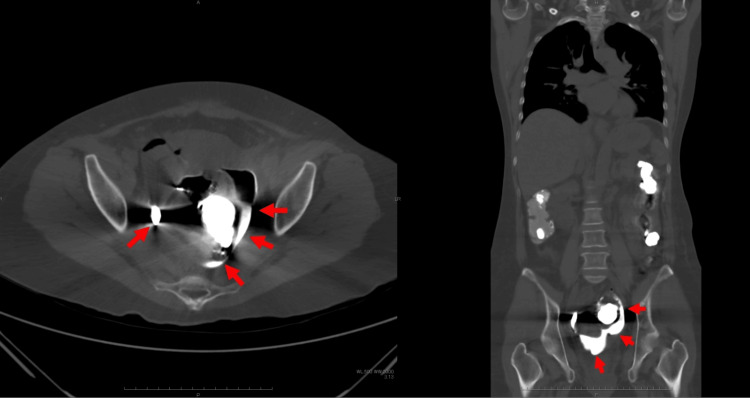
Plain abdominal CT scan A follow-up plain abdominal CT scan (axial view, left; coronal view, right) demonstrated extraluminal barium extravasation (red arrow).

A computed tomography (CT) scan was performed due to suspected gastrointestinal perforation caused by barium. Residual barium was observed extending from the sigmoid colon to the rectum. Due to halation effects, precise evaluation was challenging; however, no definite evidence of intraperitoneal free air was noted. On the third day of hospitalization, the patient experienced worsening abdominal pain and a fever of 38.5°C. Blood biochemical tests performed on the same day revealed a significant increase in CRP levels and a decrease in platelet count. A follow-up CT scan revealed the presence of extraluminal leakage of barium (Figure 1).

**Figure 2:**
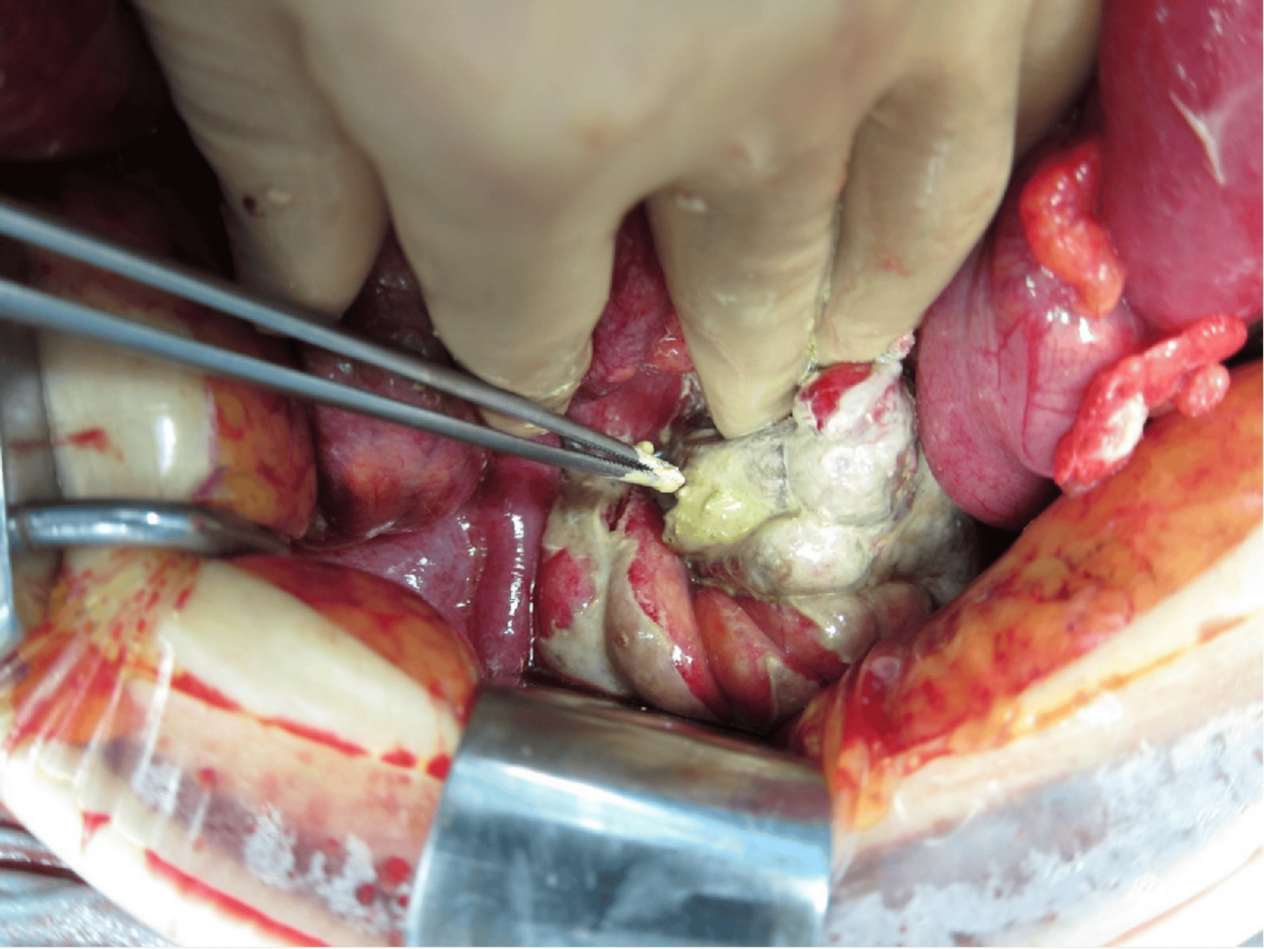
Emergency laparotomy A perforation approximately 2 cm in size was found in the upper rectum, with barium leaking into the peritoneal cavity.

An emergency laparotomy was performed with a diagnosis of peritonitis caused by gastrointestinal perforation from barium. A perforation approximately 2 cm in size was found in the upper rectum, with barium leaking into the peritoneal cavity (Figure 2). The peritoneal cavity was irrigated, and the affected portion of the rectum, including the perforation site, was resected. A colostomy was created at the proximal end (Hartmann's procedure).

**Figure 3:**
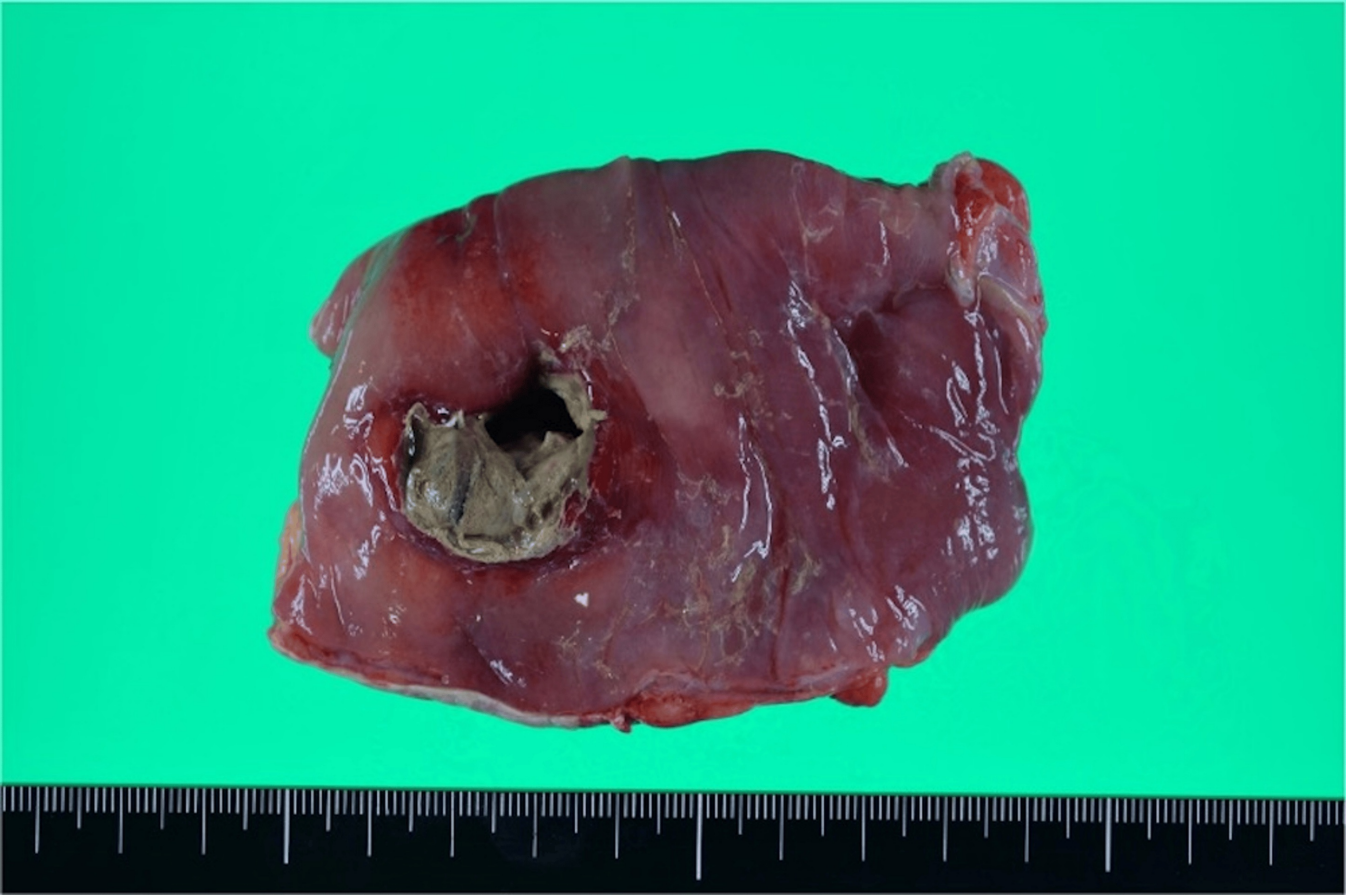
Resected specimen Necrosis and infiltration of acute inflammatory cells were observed in the intestinal tissue surrounding the perforation site. No evidence of malignancy or diverticula was found.

Pathological examination of the resected specimen revealed necrosis and infiltration of acute inflammatory cells around the perforation site. No malignancy or diverticula were identified (Figure 3).

The postoperative course was uneventful, and the patient was discharged on the 31st day after surgery. A colostomy closure was successfully performed 10 months later.

Case 2

A 74-year-old female with a history of hypertension underwent an upper gastrointestinal barium examination at a health screening facility. The next day, she developed abdominal pain, which progressively worsened, leading her to consult a previous physician. A CT scan suggested sigmoid colon perforation, and she was referred to our hospital.

The patient was 156.0 cm in height and weighed 43.0 kg. Her body temperature was 38.3°C. Blood pressure was 155/68 mmHg, and her pulse rate was 105 beats per minute. Tenderness, rebound tenderness, and muscle guarding were noted throughout the abdomen.

Blood biochemical investigations showed a decreased white blood cell count, elevated CRP levels, and increased D-dimer, indicating an inflammatory and hypercoagulable state. Detailed laboratory findings are summarized in Table 2.

**Table 2:** Serum laboratory results on admission WBC: white blood cells; RBC: red blood cells; BUN: blood urea nitrogen; AST: aspartate transferase; ALT: alanine transaminase; INR: international normalized ratio; PT: prothrombin time; APTT: partial thromboplastin time; CRP: C-reactive protein

Parameters	On admission	Reference Range
WBC	2.1	3.3-8.6 x 10^3^/uL
Neutrophils relative percent	78.6	44.0-70.0%
Lymphocytes	17.0	31.0-49.0%
Monocytes	4.4	3.0-8.0%
Eosinophils	0.0	1.0-5.0%
Basophils	0.0	0.0-3.0%
RBC	4.4	3.86-4.92 x 10^6^/uL
Hemoglobin	13.0	11.6-14.8 g/dL
Hematocrit	38.5	35.1-44.4%
Platelet count	306.0	158-348 x 10^3^/uL
PT	80.0	75-125%
INR	1.1	
APTT	27.8	25-39 seconds
D-dimer	5.5	<1.0 ug/mL
Total Protein	6.4	6.6-8.1 g/dL
Albumin	3.0	4.1-5.1 g/dL
Total Bilirubin	0.5	0.4-1.5 mg/dL
AST	24.0	13-30 U/L
ALT	14.0	7-23 U/L
Alkaline phosphatase	236.0	106-322 IFCC
Glucose	137.0	73-109 mg/dL
BUN	15.3	8-20 mg/dL
Creatinine	0.6	0.46-0.79 mg/dL
Sodium	136.0	138-145 mmol/L
Potassium	3.6	3.6-4.8 mmol/L
Chloride	105.0	101-108 mmol/L
Calcium	9.1	8.8-10.1 mg/dL
CRP	1.2	0.00-0.14 mg/dL
Lactate	2.2	<=2.0 mmol/L

**Figure 4:**
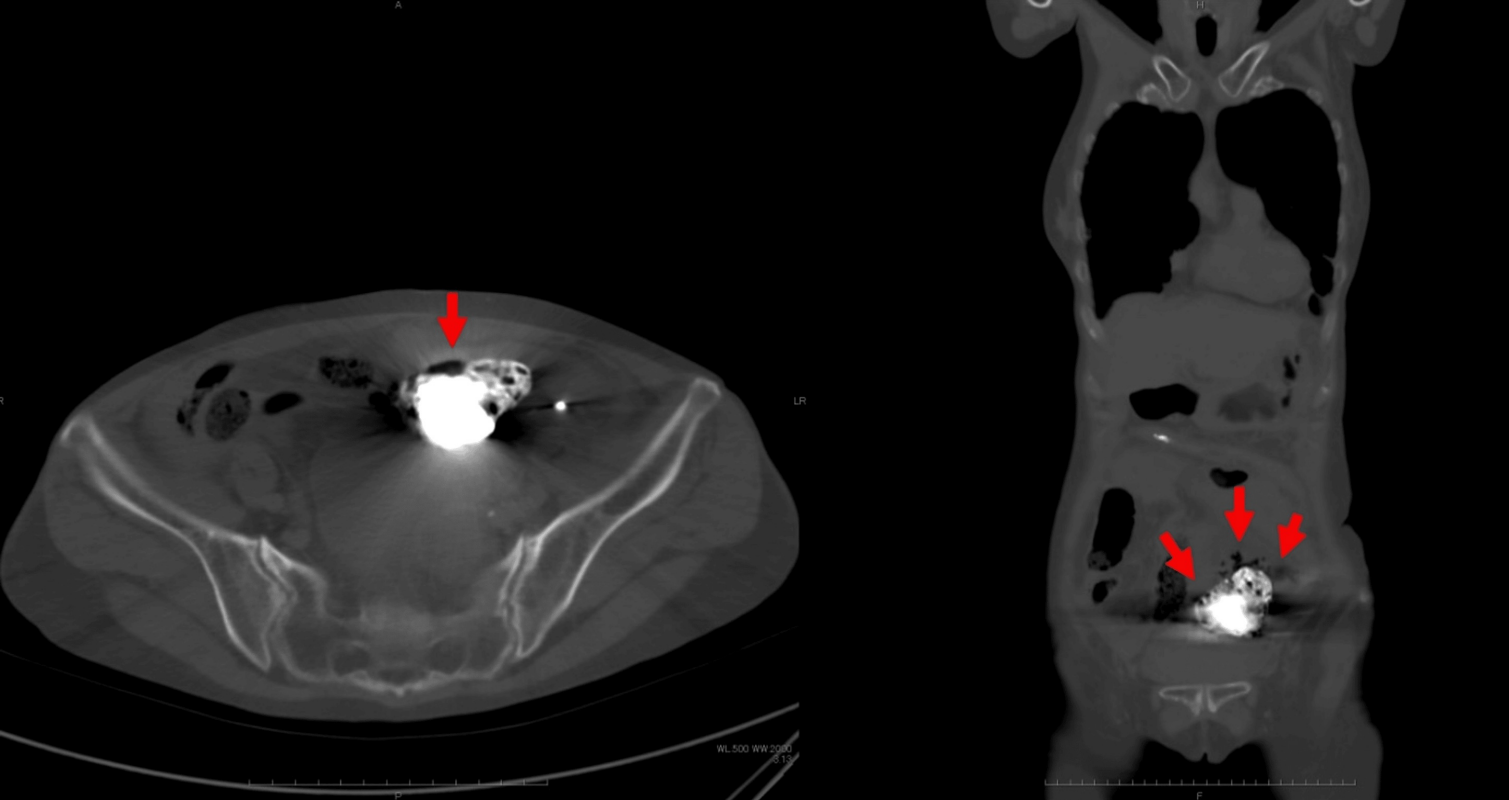
Plain abdominal CT scan A plain abdominal CT scan (axial view, left; coronal view, right) demonstrates sigmoid colon perforation with extravasation of barium contrast medium, free intraperitoneal air (red arrow), and a localized intra-abdominal abscess.

CT imaging performed by the referring physician confirmed sigmoid colon perforation due to barium. Extraluminal barium leakage, free intraperitoneal air, and a localized abscess were also identified (Figure 4).

**Figure 5:**
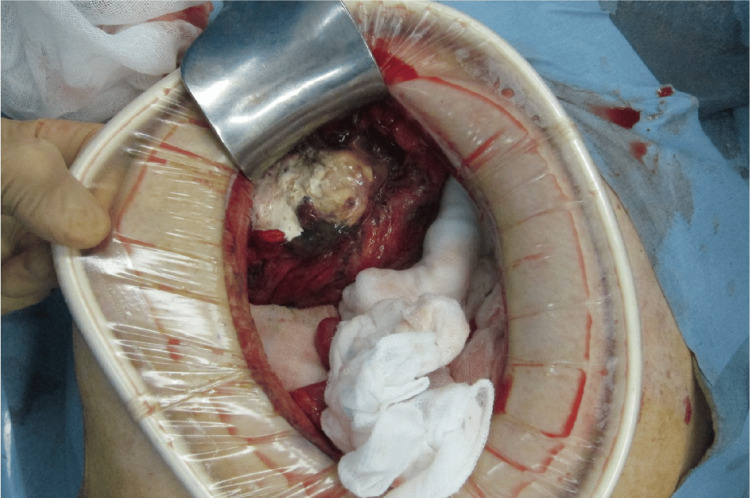
Emergency laparotomy The serosal surface of the sigmoid colon showed hematoma formation and necrotic changes, with a central perforation measuring 1 cm in diameter.

Emergency laparotomy revealed peritonitis caused by barium-induced sigmoid colon perforation. The serosal surface of the sigmoid colon exhibited hematoma formation, necrotic changes, and a central perforation measuring 1 cm in diameter. Barium extravasation into the peritoneal cavity was confirmed (Figure 5). The peritoneal cavity was thoroughly irrigated, and the affected sigmoid colon, including the perforation site, was resected. A colostomy was performed at the proximal end using Hartmann's procedure.

**Figure 6:**
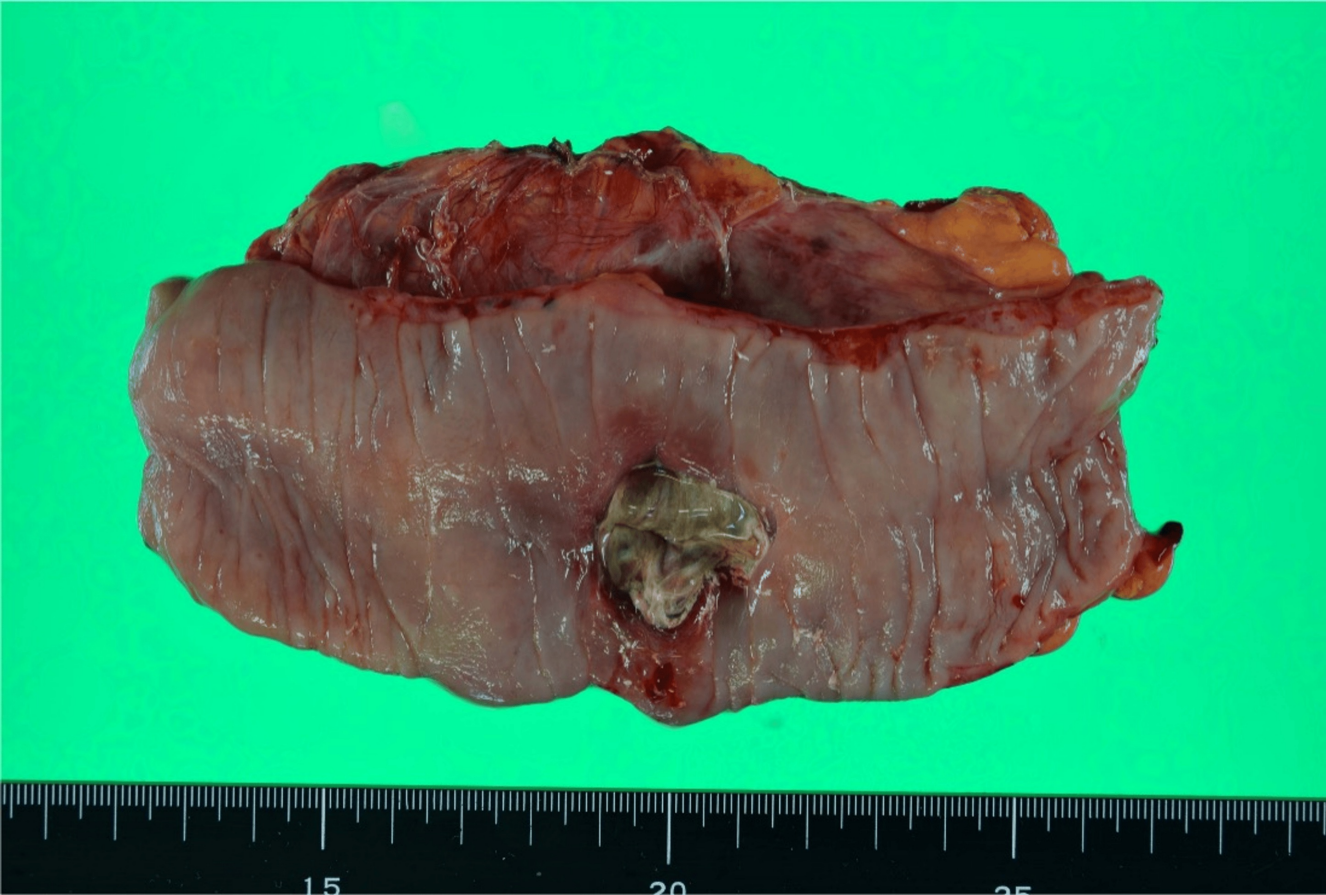
Resected specimen Severe inflammatory cell infiltration, predominantly neutrophils, was observed around the perforation site. A localized abscess was noted in part of the subserosal tissue. No evidence of malignancy or diverticula was found.

Pathological examination revealed severe inflammatory cell infiltration, predominantly neutrophils, around the perforation site. A localized abscess was present in the subserosal tissue. No malignancy or diverticula were detected (Figure 6).

The patient’s postoperative course was uneventful, and she was discharged on the 36th day after surgery. A colostomy closure was successfully performed six months later.

## Discussion

Upper gastrointestinal barium examinations, widely used in Japan's health screenings, play a critical role in detecting gastric cancer and peptic ulcers [1]. Despite their safety, rare complications such as colonic perforation, occurring in three out of 1,010,000 cases (0.0003%), have been reported [2]. Risk factors include advanced age and comorbidities, particularly in patients with diverticula or chronic constipation, where the accumulation of barium in the bowel and subsequent pressure may elevate the risk of perforation [2]. Additionally, the condition tends to be more common among elderly individuals and females, with decreased gastrointestinal flexibility and bowel stenosis due to aging acting as further risk factors [2].

There are several hypotheses regarding the mechanism of colonic perforation. Barium absorbs moisture within the intestine and hardens, making passage through the bowel difficult. It is believed that the hardened barium mass exerts strong pressure on the bowel wall, causing tears or perforation [3]. Furthermore, in cases of bowel stenosis or constipation, the intraluminal pressure tends to increase, and the retention of barium elevates the risk of perforation [5]. Most perforations occur in areas such as the sigmoid colon or rectum, which are structurally prone to increased pressure. In these areas, barium tends to accumulate, and the bowel is more susceptible to physical stretching, which can easily lead to ischemic conditions. Ischemia weakens the bowel wall, making it more prone to tears, ultimately resulting in perforation [6].

If perforation is suspected following a barium examination, patients often present with acute abdominal pain and symptoms of peritonitis. Prompt medical attention is necessary when these symptoms arise. CT is highly useful for diagnosing perforation as it allows for the identification of intraperitoneal free air and extraluminal leakage of barium, thus confirming the diagnosis. However, barium can create artifacts on CT images, potentially making it difficult to pinpoint the perforation site or detect free air in the peritoneal cavity [7]. In Case 1, the initial CT scan did not confirm gastrointestinal perforation due to the artifacts caused by barium, and the patient was placed under observation. The perforation was ultimately detected during a follow-up scan after the patient's symptoms worsened. This highlights the need for clinicians to always consider the possibility that artifacts from barium may obscure diagnosis.

When gastrointestinal perforation due to barium occurs, the inflammatory response progresses rapidly, with a high risk of infection and sepsis; therefore, emergency surgery is essential. The first-choice surgical procedures are Hartmann's procedure or a colostomy [6]. It is crucial to resect the perforated bowel segment and thoroughly irrigate the peritoneal cavity where barium has leaked. Postoperatively, strict management in an intensive care unit is required, including the administration of antibiotics and supportive therapy aimed at early recovery of bowel function. If severe inflammation progresses, postoperative recovery may take an extended period, necessitating an appropriate treatment plan based on the degree of infection.

**Figure 7:**
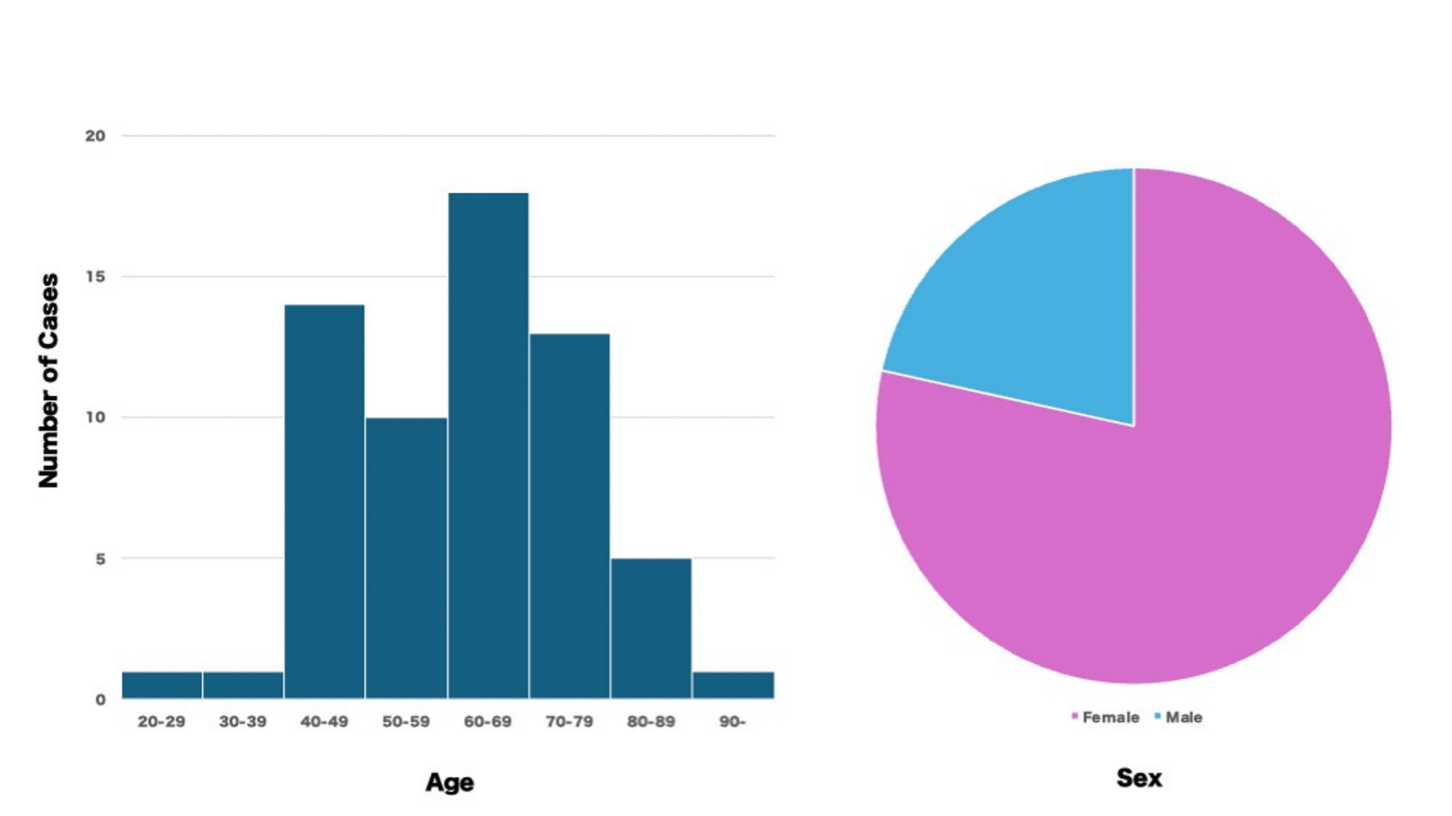
Age distribution histogram (left) and pie chart by gender (right) Image Credits: Hideo Kidogawa

We reviewed 63 reported cases of colonic perforation following upper gastrointestinal barium examinations since 2000 [4,8-38]. The average age of patients was 61.6 years (ranging from 26 to 91 years), with the majority being middle-aged to elderly individuals in their 60s. Females accounted for 77.8% of the cases (Figure 7).

**Figure 8:**
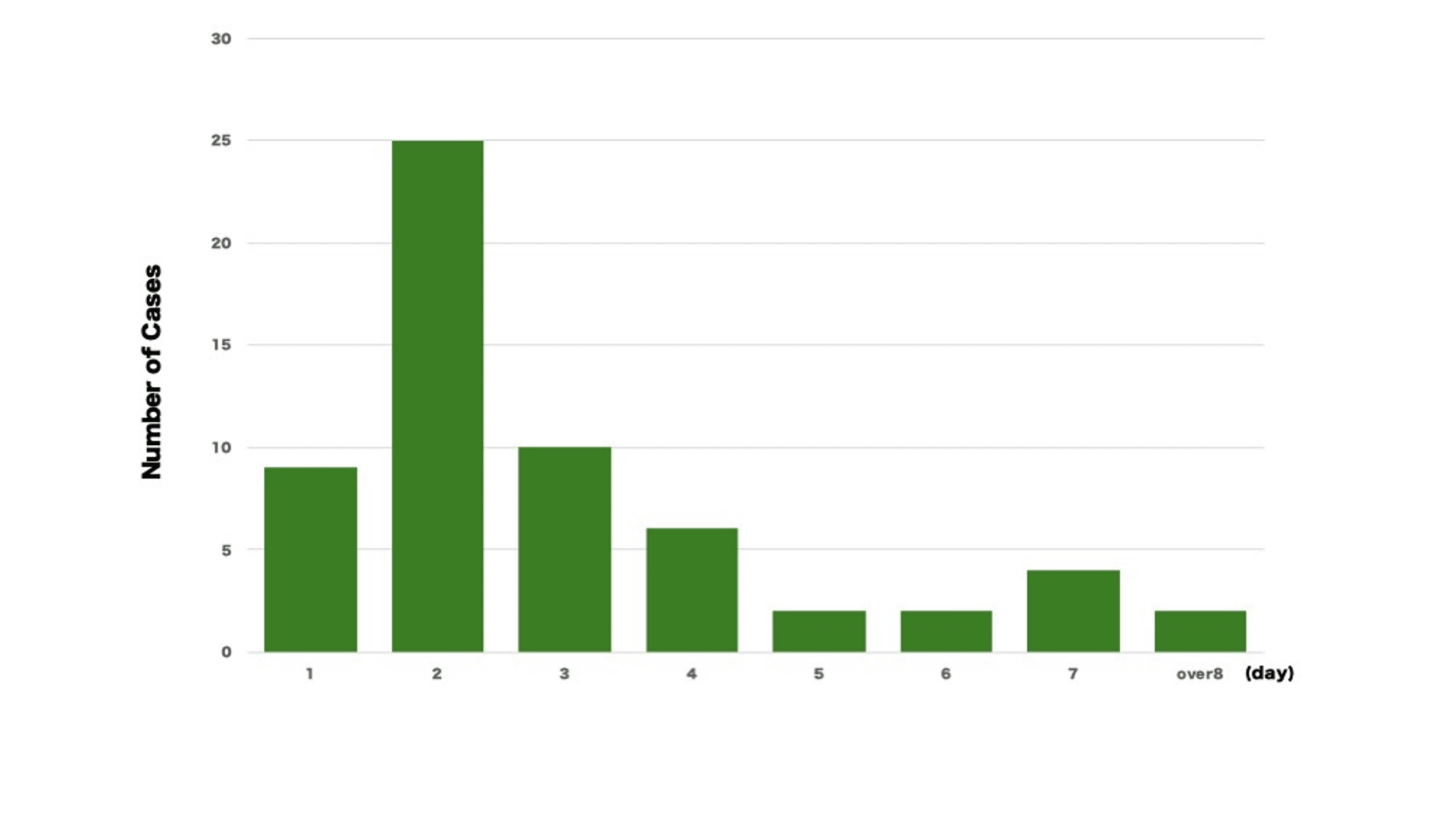
Days from examination to symptom onset Image Credits: Hideo Kidogawa

The most common time for symptom onset was within two days (Figure 8), although there was also a reported case where symptoms appeared 270 days later [9].

The most frequently affected site was the sigmoid colon (70.0%), followed by the rectum (14.3%) and the descending colon (12.7%). The most common surgical procedures were Hartmann's procedure or colostomy, comprising 77.8% of the cases, with some cases involving simple suture closure. The overall prognosis was generally favorable, though two fatalities were reported [35,38]. Many of the colostomy cases underwent stoma closure procedures several months later.

**Figure 9:**
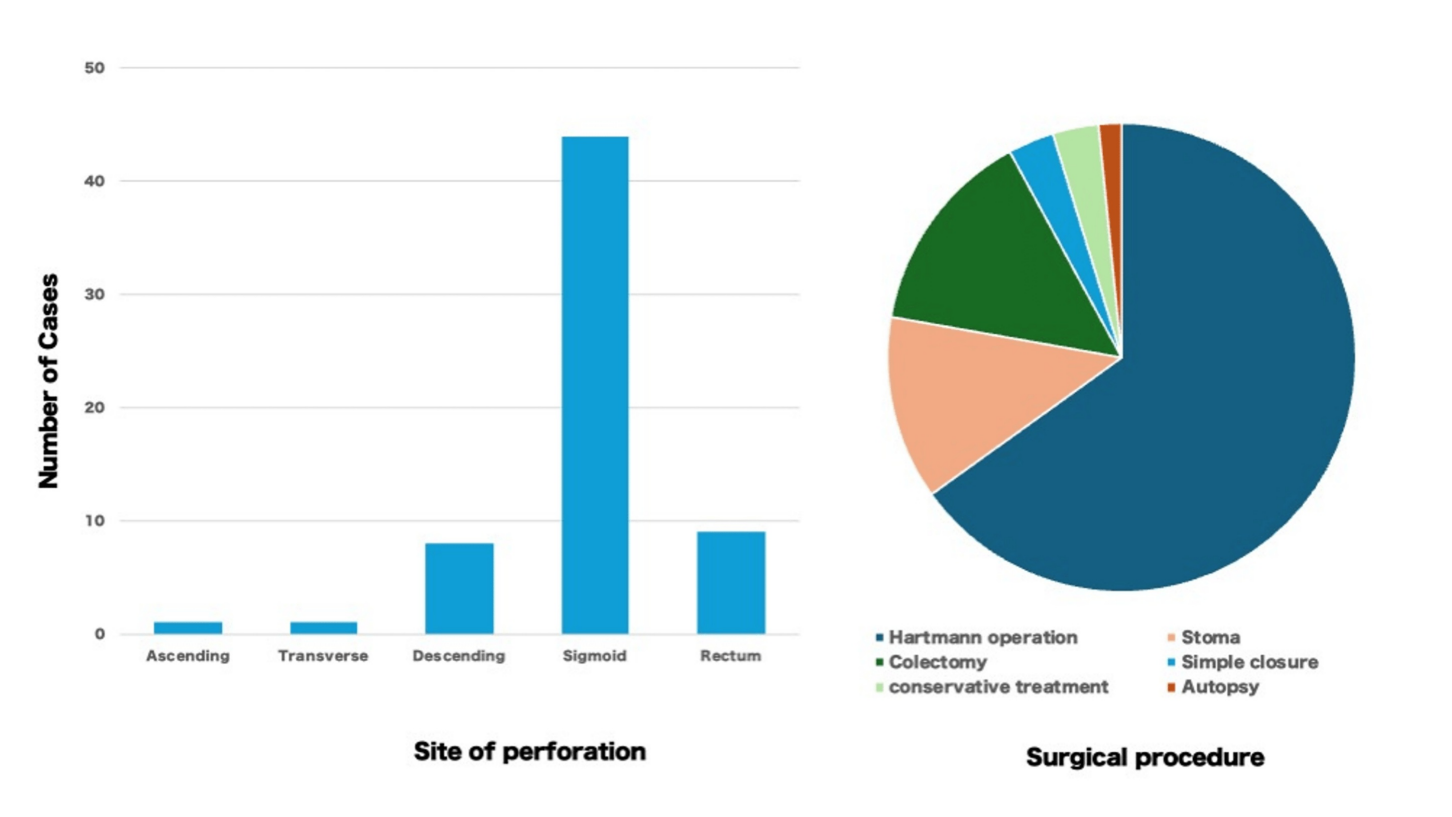
Site of perforation (left) and surgical procedure (right) Image Credits: Hideo Kidogawa

All of these reported cases were from Japan, reflecting the country's unique gastric cancer screening system [1]. In Western countries, upper gastrointestinal barium examinations are performed only in symptomatic patients and are not used for routine health screenings. Barium examinations provide an indirect method of evaluating the gastric mucosa, and their diagnostic accuracy is inferior to that of endoscopy [39]. Although considered relatively safe, gastrointestinal perforation during barium examinations, though extremely rare, can lead to fatalities and require extensive surgical procedures, imposing a significant burden on patients. Minimizing the risk of severe complications is particularly important in asymptomatic individuals undergoing routine screenings.

In recent years, endoscopic examinations have increasingly been adopted for health screenings in Japan. This report highlights the rare but serious complication of colonic perforation following upper gastrointestinal barium examinations and underscores the importance of recognizing high-risk patients to improve screening safety.

## Conclusions

We reported two cases of colonic perforation that occurred following an upper gastrointestinal series (UGIS) with barium as part of a health screening. This report provides important insights for considering the potential risks associated with UGIS in health screenings. Although the risk of gastrointestinal perforation due to barium is extremely rare, it can lead to significant complications, and early diagnosis and intervention are crucial to improving patient outcomes. It is time to consider transitioning to alternative screening methods, such as endoscopic examination.
